# Stigma, discrimination and associated determinants among people living with HIV/AIDS accessing Anti-Retroviral Therapy in Ikeja, Lagos state, Nigeria

**DOI:** 10.1038/s41598-026-37218-2

**Published:** 2026-03-14

**Authors:** Gambo Sidi Ali, Abraham Oloture Ogwuche, Alexander Idu Entonu, Kabir Adekunle Durowade

**Affiliations:** 1 Department of Public Health, College of Medicine, Ahmadu Bello University Zaria, Zaria, Nigeria; 2 Department of Medicine and Surgery, University of Ilorin, Ilorin, Nigeria; 3 Department of Community Medicine, Afe Babalola University, Ado-Ekiti, Nigeria

**Keywords:** Stigma, Discrimination, People living with HIV/AIDs, Psychology, Health care

## Abstract

Stigmatization and discrimination toward people living with HIV/AIDS (PLWHA) continue to be significant hurdles to successful treatment and social integration, especially in Nigeria. These barriers have a negative impact on mental health, deter disclosure, and reduce access to healthcare. The purpose of this study was to determine the prevalence, types, and causes of stigma and discrimination among PLWHA receiving antiretroviral therapy (ART) in Ikeja, Lagos State, Nigeria. A cross-sectional study of 400 PLWHA on ART was done at three randomly chosen treatment centers. Structured questionnaires and the Berger HIV Stigma Scale were used to collect information about individualized stigma, disclosure concerns, negative self-image, and public attitudes. The statistical study used descriptive statistics, chi-square tests, and logistic regression, with a significance level of *p* < 0.05. Overall, 37.7% of individuals reported stigma in hospital settings, 41.5% received negative attitudes from family or friends, and 48.7% felt ashamed or condemned because of their situation. Fear of revelation was widespread, with 65% refusing to declare their status. Stigma had a major impact on mental health (64.3%) and reduced ART access for 39.7% of responders. Younger age (20–39 years), poor income, HIV-positive partners, and disclosure of status were all significant predictors of stigmatization. Discrimination was highly related to occupation, gender, and socioeconomic status. Stigma and discrimination continue to be pervasive in Ikeja, limiting PLWHA access to healthcare and negatively impacting their psychological well-being. To establish a more inclusive and supportive environment, it is necessary to enhance anti-discrimination regulations, train healthcare providers, educate the public, and implement socioeconomic empowerment activities.

**Supplementary Information:**

The online version contains supplementary material available at 10.1038/s41598-026-37218-2.

## Introduction

HIV/AIDS stands as a persistent and severe health condition with widespread implications for global public health^1,2^. It was recognized as a global health problem by the World Health Organization (WHO), for over three decades since its emergence. This pandemic has inflicted devastating consequences, claiming the lives of millions globally and causing severe harm to countless others. The virus has affected over 75 million people since its onset^1^. In the year 2022 alone, 39 million individuals globally were living with HIV, and 1.3 million succumbed to AIDS-related causes^1^.

 The impact of HIV/AIDS remains particularly pronounced in the Sub-Saharan region which continues to bear the brunt of the global HIV/AIDS burden, representing two-thirds of the total cases worldwide^3^. HIV which stands for human immunodeficiency virus leads to the disease AIDS (acquired immuno- deficiency syndrome), if left untreated^4^. Presently, there are 25 million adults and children living with the virus in this region, constituting nearly 70% of the global figure. The statistics reveal an alarming scenario with approximately 1.9 million new HIV infections and 1.2 million AIDS-related deaths reported in Sub-Saharan Africa^5^. Nigeria, within this context, shoulders a significant portion of the epidemic, hosting an estimated 3.2 million individuals living with HIV, positioning the country among those with the highest HIV burden globally, second only to South Africa. In 2005, the international community embraced the goal of universal access to HIV prevention, treatment, care and support by 2010^6,7^. National HIV/AIDS programs must fortify their health systems and remove any obstacles to treatment and preventive initiatives in order to meet this objective. Nigeria has implemented several measures to stop the spread of disease^8^. People living with HIV/AIDS (PLWHAs) who have taken an HIV test and are on antiretroviral therapy (ART) have increased significantly as a result of international initiatives like the US Presidential Emergency Fund for AIDS Relief (PEPFAR) program^9^. In addition, there are many antenatal women with HIV positivity who have received ART to prevent mother to child transmission of HIV^10^. The challenges many of these global and national programs face in a multi-diverse socio-cultural society like Nigeria are the problems of stigma and discrimination(S&D)^11–14^. The issues of S&D described by Jonathan Man^15^ as the third phase of the HIV pandemic poses a serious threat to prevention and treatment. Therefore, for Nigeria to achieve her national policy on HIV/AIDS, aimed at controlling the spread of the infection and its impact, the issue of S&D needs to be addressed^8,16^. Significant research and knowledge on HIV related S&D in many ethnic and cultural settings in Nigeria, are important tool in understanding this “hidden factors” that are impediments to effective prevention and treatment. Incorporating these findings into national prevention strategies will go a long way in reducing the transmission of the virus in the population^17^.

In 2017, the demand for Anti-Retroviral Therapy (ART) in Nigeria was 1.9 million, with over 52% of those affected receiving treatment. The South-South region had the highest HIV prevalence at 3.1%, while Lagos state recorded 1.4%. Global efforts to curb HIV have significantly reduced infection rates, especially in developed countries, by extending the lives of those infected. Though no cure exists, Highly Active Anti-Retroviral Therapy (HAART) has transformed HIV from a terminal illness to a manageable chronic condition^18–20^.

Over the past decade, Nigeria has witnessed notable advancements in curbing the HIV/AIDS epidemic^21,22^. The 2019 National AIDS and Reproductive Health Survey (NARHS) demonstrated a substantial reduction in the national HIV prevalence, decreasing from a peak of 5.8% in 2001 to 3.1%^23^. This accomplishment is largely attributed to impactful interventions such as Behavior Change Programs, HIV care and support initiatives, Prevention of Mother to Child Transmission (PMTCT), and the implementation of Highly Active Anti-Retroviral Therapy (HAART)^24^. HAART treatment primarily aims to reduce the viral load to undetectable levels, fostering immune reconstitution and notable clinical improvement. Additionally, HAART has proven effective in preventing opportunistic infections^25–27^. Successful outcomes in the management of HIV/AIDS hinge significantly on adherence to HAART, as underscored by studies such as ^63 and 68^.

The reported adherence rates to ART medication among people living with HIV (PLHIV) in Nigeria vary from 44% to 98%^11,28,29^. Factors shown to be associated with good adherence include text message as reminders, patient selected treatment partners, use of pill box, age and gender. On the other hand, psychiatric morbidity negatively had adverse impacts on adherence^11^. Despite this efforts, HIV/AIDS stigma continue to re-emerge as a formidable threat, particularly among many Nigerians who lack awareness of the realities surrounding HIV and AIDS, leading to avoidance and stigmatization of individuals affected by the virus due to misconceptions about its transmission. This misguided belief suggests that one can contract the virus through association or close contact with an infected person^30^. S&D as described by various sources, represent social barriers that significantly impact the life experiences of individuals. The stigma associated with HIV and AIDS tends to marginalize people within their communities, adversely affecting the overall quality of life for PLHIV. Stigma is often synonymous with social disgrace. Research indicates that individuals reporting high levels of stigma are more than four times likely to experience limited access to healthcare. Moreover, HIV and AIDS-related stigma can give rise to discrimination, such as restrictions on travel, healthcare facility usage, employment opportunities, and social interactions for PLHIV^13,31,32^.

Although there have been few studies as regard stigma and discrimination against individuals living with HIV/AIDS^33–56^, it remains prevalent, impacting their access to care and quality of life. This research aims to comprehensively explore the knowledge, prevalence, types and determinants of stigma and discrimination among people accessing Anti-retroviral Therapy (ART) in Ikeja, Lagos State, Nigeria.

This research aims to fill a gap in the literature by exploring the relationship between stigma, discrimination, and factors affecting access to anti-retroviral therapy (ART) for people living with HIV/AIDS (PLWHA) in Ikeja, Lagos State, Nigeria. The geographical scope is limited to Ikeja, a prominent urban area, allowing for an in-depth exploration within a defined locale. It seeks to provide practical insights for the local healthcare sector, identifying cultural, socioeconomic, and healthcare-related influences that impact PLWHA’s well-being. The findings could guide policymakers and healthcare professionals in developing inclusive interventions to reduce stigma and improve healthcare practices. Additionally, the study aims to empower both PLWHA and the wider community by increasing public understanding and contributing to global efforts against HIV-related discrimination.

### Theoretical framework

People living with HIV/AIDS (PLWHA) can be better understood through the perspective of Erving Goffman’s theory of Stigma (1963). According to Goffman, stigma is a social process that discredits and diminishes a person who possesses a “undesired differentness” from a whole individual to a contaminated, rejected one^57^. He distinguished three types of stigma: (i) stigma related to physical defects, (ii) stigma related to personal character flaws, and (iii) stigma related to tribes or groups^57^. All three factors are present in HIV/AIDS-related stigma: affiliation with marginalized populations, moral assessments of sexual activity or drug use, and evident sickness and side effects. According to this paradigm, stigma is a relational and structural phenomenon that influences social interactions, health-related behaviors, and resource accessibility in addition to being an individual experience^58^.

## Methodology

This study that was conducted in Lagos State in January 2024, focused on Ikeja, a major economic and healthcare hub. Despite being the smallest state by landmass, Lagos had a population of 12.7 million in 2019, with Ikeja being home to over 470,200 people^33,59^. Overcrowding, rural-urban migration, and low-income earners characterize the state. Ikeja’s diverse urban-suburban population and its healthcare infrastructure, including several busy Anti-Retroviral Therapy (ART) clinics, provide an ideal backdrop to examine stigma and discrimination among people living with HIV/AIDS (PLWHA). The study aims to highlight the socio-economic and healthcare challenges affecting access to ART services in this region.

A cross-sectional hospital-based study conducted at the Antiretroviral Therapy Centre (ATC) was employed. Among all adult clients on ART regimen, accessing treatment at any HAART clinic in Ikeja, Lagos state. Using standard methods and procedures^60^.

### Inclusion criteria

- Adult HIV patients 18 years and above.
- Patients with confirmed HIV-positive status who had received Anti-Retroviral drugs for at least 1 week prior to the study. This was to allow only established HAART clinic users in the study.
- Individuals accessing Anti-retroviral Therapy (ART) services in any HAART clinic within Ikeja, Lagos state.

### Exclusion criteria

- Pregnant women.
- Terminally ill/debilitated patients.
- Patients on admission.

Participation was open to all adult PLWHA (≥ 18 years) who were registered and receiving ART at the chosen sites. There were two steps involved in applying the exclusion criterion. First, during regular ART appointments, potential respondents were contacted after being found in clinic records. Second, the attending healthcare providers were consulted during the eligibility screening process. Patients with advanced AIDS-related comorbidities, physically incapacitating conditions, or those receiving palliative/end-of-life care were considered terminally ill and were not recruited into the study. Furthermore, those who were too sick to finish the questionnaire, had cognitive impairments, or were incapable of giving informed consent were excluded. This made sure that only stable patients could participate in the study in a meaningful way.

A sample size of 400 was selected using the fischer formula. A simple random sampling technique was used. Out of seven known ART centers, three hospitals were selected using simple random sampling. The names of all seven centers were written on separate slips of paper, placed in a container, and three slips were drawn randomly to select Lagos State University Teaching Hospital Ikeja, 661 Nigerian Air Force Hospital Ikeja, and Primary Health Centre Ogba. For patient selection, a list of all PLWHAs undergoing ART at these hospitals was obtained using a random number generator, 400 patients were chosen from these lists to participate in the study, ensuring an unbiased and representative sample.

Structured questionnaires (see appendix 2) was administered to individuals accessing ART in Ikeja to gather quantitative data on their experiences of stigma, discrimination, and related factors. These instruments include validated scales or items measuring stigma levels, discrimination experiences, and healthcare access barriers. The socio-demographic details of the participants was recorded using a pretested questionnaire schedule. Social stigma related to HIV was assessed using the Berger HIV Stigma Scale. During the process of patient interaction, queries/questions regarding HIV/AIDS/ART was clarified and solved. Any additional open-ended responses of the subjects was noted, without further probing.

The Berger Stigma Scale measures HIV-related stigma across four domains: Personalized Stigma, Disclosure Concerns, Negative Self Image, and Public Attitudes, totaling 40 items. Scores range from 40 to 160, with higher scores indicating greater stigma^61,62^. The scale has strong reliability (coefficients alphas of 0.90–0.93 for subscale and 0.96 overall reliability). These findings validate the scale’s suitability for use among PLWHA in Ikeja, Lagos, and show strong internal consistency. It takes 15–25 min to complete. Knowledge of HIV was assessed through a separate scoring system, with scores over 60% classified as good knowledge.

### Ethical approval/consent/regulations

Ethical approval was obtained from the Lagos state university teaching hospital Health Research and Ethics Committee with the Ref.No LREC/06/10/2499. Informed consent was obtained from each participants. Consent was explained in local dialect where applicable. Participants were informed of their right to withdraw from the study at any time without penalty. Research assistants were trained to ensure confidentiality, and no personal identifiers were included in the data to prevent stigma. (see appendix 1). All methods were performed in accordance with the relevant guidelines and regulations.

Data was analyzed using STATA version 11.0, with statistical tests like Chi-squared, t-statistic, and logistic regression. A p-value of < 0.05 was considered significant. Univariate analysis presented frequencies, percentages, means, and standard deviations. Knowledge scores were graded, and associations between stigma, discrimination, and ART accessibility were tested using Chi-square and logistic regression, with significance set at *p* < 0.05.

## Result

A total of 409 questionnaires were given out and 400 returned indicating a 98% response rate. Univariate analysis is expressed as frequency tables, mean and standard deviation while bivariate and multivariate was presented in tables.

The first section of the finding’s presentation covered the respondents’ demographic and socioeconomic details.

**Table 1:** Demographic characteristics of respondents. *N* = 400.

Variable	Frequency	Percentage (%)
**Age** (in years)		
< 20	6	1.5
20–29	105	26.3
30–39	110	27.5
40–49	107	26.7
≥ 50	72	18.0
**Mean Age ± SD**	**39 ± 11**	
**Gender**		
Female	201	50.3
Male	199	49.7
**Marital status**		
Single	147	36.8
Married	195	48.8
Separated/Divorced	31	7.7
Widowed	27	6.7
**Ethnicity**		
Yoruba	120	30.0
Hausa	43	10.7
Igbo	121	30.3
Others	116	29.0
**Religion**		
Christianity	308	77.0
Islam	92	23.0
**Education status**		
None	8	2.0
Primary	11	2.8
Secondary	87	21.7
Tertiary	294	73.5
**Occupation**		
Civil servant	136	34.0
Artificer	20	5.0
Trader	168	42.0
Student	28	7.0
Others	48	12.0
**Average monthly income (₦)**		
< 50,000	113	28.3
50,000–100,000	71	17.7
100,001–500,000	119	29.7
> 500,000	97	24.3
**Average monthly income ± SD**	**301568.1 ± 318389.2**	
**HIV status Disclosure**		
Undisclosed	215	53.7
Disclosed	185	46.3
**Partner’s HIV status**		
Positive	171	42.8
Negative	110	27.5
Unknown	119	29.7
**Duration on ART**		
< 5	211	52.7
≥ 5	189	47.3
**Mean duration on ART ± SD** (in years)	**5.3 ± 5**	
**Area of Residence**		
Within Ikeja	148	37.0
Outside Ikeja LGA	173	43.3
Outside Lagos State	79	19.7

From Table 1, more than a quarter of respondents were in the 20-29years (26.3%), 30-39years (27.5%), and 40-49years (26.7%) with a mean of 39 ± 11. Gender distribution shows a near-equal split with 50.3% female and 49.7% male. As regards the level of education, more than two thirds of participants (73.5%) have completed university education, with 2.0% having no formal education. The average monthly income of the participants varies; smaller proportion earn between ₦50,000 and ₦100,000 (17.7%), while the bulk earn between ₦100,000 and ₦500,000 (29.7%). The monthly average income is roughly ₦301,568 ± ₦318,389.

Concerning HIV status disclosure, more than one third of participants (46.3%) have declared their status, compared to over half (53.75%) who have not. For partners’ HIV status more than one third (42.75%) of the participants reported having an HIV-positive spouse. With a mean of 5.3 ± 5 years, the participants’ length of time on ART reveals more than half of participants (52.7%) have been on therapy for less than five years.

### Awareness and knowledge of stigma and discrimination

**Table 2:** Assessment of level of awareness and knowledge regarding stigma and discrimination. *N* = 400.

Variable	Yes (%)
Are you aware of what stigma related to HIV/AIDS means?	359(89.7)
Have you received information regarding discrimination against PLWHAs?	232(58.0)
Do you know about support services available to address stigma and discrimination faced by PLWHAs?	187(46.7)
Have you participated in educational programs related to HIV/AIDS stigma and discrimination?	184(46.0)
Are you aware that reducing stigma could improve healthcare access for PLWHAs?	317(79.3)
**Knowledge**	
Good	**160(40.0)**
Poor	**240(60.0)**

Table 2 Among the respondents, 89.7% were aware of what stigma related to HIV/AIDS means, indicating high awareness levels. However, only 46.7% knew about support services available to address stigma and discrimination faced by PLWHAs. Furthermore, 79.3% were aware that reducing stigma could improve healthcare access for PLWHAs. Despite high awareness, more than half (60.0%) of respondents had poor knowledge overall.

### Prevalence of stigma and discrimination

**Table 3:** Determination of level of prevalence of stigma and discrimination encountered by respondents. *N* = 400.

Variable	Yes
Have you ever been treated differently by healthcare providers due to your HIV status?	155(38.7)
Have you encountered negative attitudes or behaviors from family or friends because of your HIV status?	166(41.5)
Have you ever felt ashamed or judged because of your HIV status?	195(48.7)
Do you believe there is widespread discrimination against PLWHAs in Ikeja?	192(48.0)
Have you personally experienced verbal abuse or insults related to your HIV status?	127(31.7)
**Prevalence**	
High	**81(20.3)**
Low	**319(79.7)**

From Table 3 above, more than a third of participants (38.7%) reported being treated differently by healthcare providers due to their HIV status. Negative attitudes or behaviors from family or friends were encountered by 41.5% of participants, 48.7% felt ashamed or judged because of their HIV status, and 48.0% believed there is widespread discrimination against PLWHAs in Ikeja. Personal experiences of verbal abuse or insults related to HIV status were reported by less than a third (31.7%) of respondents.

The prevalence of stigma and discrimination was generally low, with more than two third (79.7%) of respondents classified as experiencing low prevalence. This classification was based on scoring and grouping responses into low and high prevalence categories.

### Forms of stigma and discrimination

**Table 4:** Shows the different forms of stigma and discrimination experienced by respondents. *N* = 400.

Variable	Yes (%)
Have you experienced stigma while accessing healthcare services for HIV/AIDS	151(37.7)
Have you encountered discrimination in educational or workplace settings due to	119(29.7)
Do you perceive media or societal attitudes contribute to stigma associated with HIV/AIDS	208(52.0)
Do religious or cultural beliefs influence the treatment of PLWHAs in your community	182(45.5)
Have you witnessed/experience instances where PLWHAs were excluded from community	129(32.3)
Do you believe poverty or socioeconomic status influences how PLWHAs are treated	224(56.0)
Have you experienced stigma or discrimination due to gender or sexual orientation	164(41.0)
Do healthcare provider attitudes affect stigma faced by PLWHAs	235(58.7)
Do you think education level or awareness impacts how people treat PLWHAs in Ikeja	278(69.5)
Are political or governmental policies contributing to discrimination against PLWHAs	209(52.3)
Do you perceive the community in Ikeja to hold negative attitudes towards PLWHAs	174(43.5)
Have you observed community-based programs aimed at reducing stigma against PLWHAs	174(43.5)
Do you believe media representations of HIV/AIDS contribute to negative perception	224(56.0)
Do you think healthcare provider attitudes affect PLWHAs’ willingness to seek help	268(67.0)
Do PLWHAs in Ikeja face challenges accessing ART and necessary healthcare service	159(39.7)
Have you or others encountered barriers/difficulties accessing healthcare due to your HIV status?	163(40.7)
Are PLWHAs in Ikeja comfortable disclosing their HIV status to others?	89(22.3)
Does fear of stigma or discrimination influence the decision to disclose HIV status?	260(65.0)
Have you observed changes in your mental health or self-esteem because of your HIV status?	219(54.7)
Do you believe stigma significantly affects the mental well-being and social interactions of PLHVAs?	257(64.3)
Do you think poverty amplifies the stigma experienced by PLWHAs in Ikeja?	248(62.0)
Have you or others encountered barriers/difficulties accessing healthcare due to HIV/AIDS-related stigma?	163(40.7)
Does fear of stigma or discrimination influence the decision to disclose HIV status?	260(65.0)
Have you observed changes in your mental health or self-esteem because of your HIV status?	219(54.7)
Do you believe stigma significantly affects the mental well-being and social interactions of PLWHAs?	257(64.3)
Do you think poverty amplifies the stigma experienced by PLWHAs in Ikeja?	248(62.0)
Have you noticed differences in the treatment of PLWHAs based on socioeconomic status?	196(49.0)
Can education and awareness programs substantially reduce stigma and discrimination related to HIV/AIDS?	283(70.7)
Have educational campaigns in Ikeja shown any impact on reducing stigma against PLWHAs?	232(58.0)
Have been gossiped about	96(24.0)
Verbally insulted/harassed or threatened	82(20.5)
Husband, spouse, or other household members have been discriminated against	75(18.7)
Sexual rejection	125(31.3)
Excluded from social gatherings	61(15.3)
Excluded from family activities	65(16.3)
Discriminated against by other PLWHAs	52(13.0)
Excluded from religious activities	42(10.5)

From Table 4, two third (67.0%) of respondents believe that healthcare provider attitudes affect PLWHAs’ willingness to seek help, highlighting a crucial factor in stigma and discrimination, 69.5% think that education and awareness impact how people treat PLWHAs in Ikeja. The perception that poverty or socioeconomic status influences the treatment of PLWHAs is shared by more than half (56.0%) of respondents, while the fear of stigma or discrimination affecting the decision to disclose HIV status was noted by 65.0% of participants. Furthermore, about two third (64.3%) of participants believe that stigma significantly impacts the mental well-being and social interactions of PLWHAs and 70.7% agree that education and awareness programs can substantially reduce stigma and discrimination related to HIV/AIDS.

**Table 5:** Subscale of stigma distribution with Gender.

Subscale	Mean Score	Male	Female	*P* (Independent t-test)
Personalized stigma	2.46 ± 2.04	2.29 ± 2.04	2.63 ± 2.02	0.0946
Disclosure concerns	1.43 ± 0.62	1.42 ± 0.60	1.44 ± 0.64	0.7391
Negative self-image	1.68 ± 1.02	1.61 ± 1.07	1.74 ± 0.96	0.1748
Public attitude	6.32 ± 4.43	6.48 ± 4.64	6.15 ± 4.42	0.4760

Table 5 demonstrated the mean scores for the various HIV/AIDS stigma subscales among participants who were male and female, as well as the findings of an independent t-test comparing these scores. 2.46 ± 2.04 was the mean score for personalized stigma. Although the mean score of females was slightly higher (2.63 ± 2.02) than that of males (2.29 ± 2.04), there was no statistically significant difference between the two groups (*p* = 0.0946).

Participants’ average score for disclosure concerns was 1.43 ± 0.62. Regarding these issues, there was no discernible difference between the male and female participants (1.42 ± 0.60 and 1.44 ± 0.64, respectively; *p* = 0.7391).

The mean score for having a poor self-image was 1.68 ± 1.02. The mean score of females was 1.74 ± 0.96, somewhat higher than that of males (1.61 ± 1.07), although this difference was not statistically significant (*p* = 0.1748). The mean score for public attitude was 6.32 ± 4.43. Regarding public attitude, there was no statistically significant difference between males (6.48 ± 4.64) and females (6.15 ± 4.42) (*p* = 0.4760).

All things considered, these findings imply that there were no appreciable gender disparities in the participants’ mean scores for personalized stigma, disclosure worries, negative self-image, or public opinion.

### Interpretation of stigma subscale correlations

**Table 6:** Correlation between the different subscales of stigma.

Stigma subscale	Personalized stigma	Disclosure concerns	Negative self-image	Public attitude
Personalized stigma	1			
Disclosure concerns	0.0296	1		
Negative self-image	0.5838	0.1195	1	
Public attitude	0.8012	0.0554	0.4698	1

Table 6 revealed significant relationships between stigma subscales in the study. There is a moderate positive correlation between personalized stigma and negative self-image at 0.5838, indicating that higher levels of personalized stigma are associated with more negative self-perceptions. A strong positive correlation of 0.8012 exists between personalized stigma and public attitude, suggesting that personal stigma is closely linked to perceptions of societal attitudes. Disclosure concerns show a weak positive correlation with personalized stigma at 0.0296, and a weak positive correlation with negative self-image at 0.1195. The strongest correlation is between personalized stigma and public attitude. Meanwhile, public attitude and negative self-image have a moderate positive correlation of 0.4698, while disclosure concerns and public attitude show a weak correlation of 0.0554.

### Factors contributing to experience of stigma and discrimination

**Table 7:** Association of socio-demographic parameters of study participant and stigmatization.

Variable	Stigmatization			χ²	*P* value
	High (%)	Low (%)	Total (100%)		
**Age**					
< 20	2(33.3)	4(66.7)	6		
20–29	34(32.4)	71(67.6)	105		
30–39	47(42.7)	63(57.3)	110	3.33	0.504
40–49	35(32.7)	72(67.3)	107		
>=50	25(34.7)	47(65.3)	72		
**Average monthly income (#)**					
< 50,000	105(92.9)	8(7.1)	113		
50,000–100,000	38(53.5)	33(46.5)	71	290.74	< 0.001
100,001–500,000	0(0.0)	119(100.0)	119		
> 500,000	0(0.0)	97(100.0)	97		
**Gender**					
Male	77(38.7)	122(61.3)	199	1.49	0.222
Female	66(32.8)	135(67.2)	201		
**Marital Status**					
Single	55(37.4)	92(62.6)	147		
Married	70(35.9)	125(64.1)	195		
Separated/Divorced	10(32.3)	21(67.7)	31	0.78	0.853
Widowed	8(29.6)	19(70.4)	27		
**Partner’s HIV status**					
Positive	74(43.3)	97(56.7)	171		
Negative	30(27.3)	80(72.7)	110	8.12	0.017
Unknown	39(32.8)	80(67.2)	119		
**Ethnicity**					
Yoruba	45(37.5)	75(62.5)	120		
Hausa	14(32.6)	29(67.4)	43	0.97	0.808
Igbo	40(33.1)	81(66.9)	121		
Others	44(37.9)	72(62.1)	116		
**Religion**					
Christianity	106(34.4)	202(65.6)	308	1.04	0.308
Islam	37(40.2)	55(59.8)	92		
**Educational status**					
None	3(37.5)	5(62.5)	8		
Primary	4(36.4)	7(63.6)	11	6.57	0.087
Secondary	21(24.1)	66(75.9)	87		
Tertiary	115(39.1)	179(60.9)	294		
**HIV status disclosure**					
Undisclosed	76(35.4)	139(64.6)	215		
Disclosed	67(36.2)	118(63.8)	185	0.03	0.857
**Occupation**					
Civil servant	57(41.9)	79(58.1)	136		
Artificer	5(25.0)	15(75.0)	20		
Trader	54(32.1)	114(67.9)	168	4.21	0.379
Student	10(35.7)	18(64.3)	28		
Others	17(35.4)	31(64.6)	48		
**Area of Residence**					
Within Ikeja LGA	51(34.5)	97(65.5)	148		
Outside Ikeja LGA	58(33.5)	115(66.5)	173	2.31	0.316
Outside Lagos State	34(43.0)	45(57.0)	79		
**Duration on ART (in years)**					
< 5	75(35.6)	136(64.4)	211	0.01	0.928
≥ 5	68(36.0)	121(64.0)	189		

Table 7 shows that the partner’s HIV status was found to be a significant variable in the stigmatization analysis. With *p* = 0.017 and χ² = 8.12, there was clear statistical significance. This implies that a person’s ability to avoid stigmatization is significantly influenced by the HIV status of their partners.

Additionally, there was an association between respondents’ average monthly income and stigmatization (χ² = 290.74, *p* < 0.001, indicating that socioeconomic position is a key factor influencing stigmatization.

However, a number of factors had no statistically significant association with stigmatization. The χ² = 3.33 and the *p* = 0.504 for age did not indicate an association.

The following factors did not significantly differ (*p* > 0.050): gender, marital status, ethnicity, religion, educational status, disclosure of HIV status, occupation, place of residence, and duration on ART.

**Table 8:** Association of socio-demographic parameters of study participants and Discrimination.

Variable	Discrimination			χ²	*P* value
	High (%)	Low (%)	Total (100%)		
**Age**					
< 20	0(0.0)	6(100.0)	6		
20–29	9(8.6)	96(91.4)	105		
30–39	12(10.9)	98(89.1)	110	1.50	0.826
40–49	12(11.2)	95(88.8)	107		
≥ 50	9(12.5)	63(87.5)	72		
**Average monthly income** (#)					
< 50,000	20(17.7)	93(82.3)	113		
50,000–100,000	16(22.5)	55(77.5)	71	33.06	< 0.001
100,001–500,000	0(0.0)	119(100.0)	119		
> 500,000	6(6.2)	91(93.8)	97		
**Gender**					
Male	18(9.0)	181(91.0)	199	0.89	0.345
Female	24(11.9)	177(88.1)	201		
**Marital Status**					
Single	12(8.2)	135(91.8)	147		
Married	23(11.8)	172(88.2)	195	2.52	0.471
Separated/Divorced	5(16.1)	26(83.9)	31		
Widowed	2(7.4)	25(92.6)	27		
**Partner’s HIV status**					
Positive	31(18.1)	140(81.9)	171		
Negative	4(3.6)	106(96.4)	110	18.80	< 0.001
Unknown	7(5.9)	112(94.1)	119		
**Ethnicity**					
Yoruba	17(14.2)	103(85.8)	120		
Hausa	4(9.3)	39(90.7)	43	3.79	0.285
Igbo	8(6.6)	113(93.4)	121		
Others	13(11.2)	103(88.8)	116		
**Religion**					
Christianity	28(9.1)	280(90.9)	308	2.83	0.093
Islam	14(15.2)	78(84.8)	92		
**Educational status**					
None	2(25.0)	6(75.0)	8		
Primary	0(0.0)	11(100.0)	11		
Secondary	9(10.3)	78(89.7)	87	3.08	0.379
Tertiary	31(10.5)	263(89.5)	294		
**Status disclosure**					
Undisclosed	16(7.4)	199(92.6)	215		
Disclosed	26(14.1)	159(85.9)	185	4.63	0.031
**Occupation**					
Civil servant	18(13.2)	118(86.8)	136		
Artificer	6(30.0)	14(70.0)	20		
Trader	13(7.7)	155(92.3)	168	13.82	0.008
Student	0(0.0)	28(100.0)	28		
Others	5(10.4)	43(89.6)	48		
**Area of Residence**					
Within Ikeja LGA	21(14.2)	127(85.8)	148		
Outside Ikeja LGA	11(6.4)	162(93.6)	173	5.69	0.058
Outside Lagos State	10(12.7)	69(87.3)	79		
**Duration on ART**					
< 5	23(10.9)	188(89.1)	211	0.08	0.782
≥ 5	19(10.0)	170(90.0)	189		

Table 8 shows that average monthly income has an association with discrimination among the factors analyzed. With *p* < 0.001 and χ² = 33.06 there was clear statistical significance. This implies that people’s experiences of discrimination are significantly influenced by their financial levels.

The presence of HIV in a partner was also significantly associated with discrimination (χ² = 18.80, *p* < 0.001). This suggested that a person’s ability to avoid discrimination is significantly influenced by their partner’s HIV status. Another variable that showed statistical significance in relation to discrimination was status disclosure (χ² = 4.63, *p* = 0.031). This suggests that a person’s chance of facing discrimination is greatly impacted by whether they disclose their HIV status.

It was also found that discrimination was associated with occupation (χ² = 13.8243, *p* = 0.008) indicating that occupational status has an effect on experiences of discrimination.

However, several factors (including age, marital status, religion, ethnicity, educational attainment, place of residence, and duration on ART) did not show statistically significant differences from one another when it came to discrimination (p value > 0.05).

**Table 9:** Association of socio-demographic parameters of study subjects with stigmatization (Logistic regression analysis) *N* = 400.

Variable	Stigmatization	
	OR [95% Conf. Interval]	*P* > z
**Age (years)**		
< 20	Reference	
20–29	50.41(1.66185–1529.633)	0.024
30–39	44.82(1.607253–1249.628)	0.025
40–49	7.56(0.335789–170.0604)	0.203
> 50	5.92(0.256746–136.8447)	0.267
**Gender**		
Female	Reference	
Male	1.22(0.451968–3.295689)	0.694
**Marital Status**		
Single	Reference	
Married	2.30(0.494824–10.70581)	0.288
Divorced	2.14(0.313285–14.66395)	0.437
Widowed	1.68(0.088629–31.76929)	0.730
**Ethnicity**		
Yoruba	Reference	
Hausa	0.10(0.016882–0.619667)	0.013
Igbo	1.57(0.356462–6.931393)	0.550
Others	1.11(0.310046–3.975394)	0.872
**Religion**		
Christianity	Reference	
Islam	2.34(0.56546–9.66315)	0.241
**Educational Status**		
None	Reference	
Primary	0.06(0.000758–4.869785)	0.21
Secondary	0.28(0.007398–10.97426)	0.500
Tertiary	0.38(0.010955–12.88153)	0.587
**Occupation**		
Civil servant	Reference	
Artificer	0.83(0.024666–28.21668)	0.920
Trader	0.72(0.08047–6.462311)	0.770
Student	0.42(0.123071–1.40783)	0.159
**Place of residence**		
Within Ikeja	Reference	
Outside Ikeja LGA	1.39(0.478736–4.049149)	0.543
Outside Lagos State	4.75(1.085868–20.8087)	0.039
**HIV status disclosure**		
Undisclosed	Reference	
Disclosed	4.37(1.406066–13.56713)	0.011
**Partner’s Status**		
Positive	Reference	
Negative	0.61(0.18053–2.091202)	0.436
Unknown	0.70(0.138353–3.495726)	0.659
**Years you have been on ART**		
< 5	Reference	
≥ 5	0.88(0.276574–2.793859)	0.827
**Average monthly Income(₦)**		
< 50,000	Reference	
50,000–100,000	0.03(0.007962–0.110472)	< 0.001
100,001–500,000		
> 500,000		

Table 9 highlights several statistically significant variables associated with stigmatization. Individuals aged 20–29 years have a significantly higher likelihood of experiencing stigmatization with an odds ratio (OR) of 50.41 (95% CI: 1.66–1529.63, *P* = 0.024), and those aged 30–39 years have an OR of 44.82 (95% CI: 1.61–1249.63, *P* = 0.025). Ethnicity also plays a role, as Hausa individuals are less likely to experience stigmatization with an OR of 0.10 (95% CI: 0.02–0.62, *P* = 0.013). Those living outside Lagos State have an increased likelihood of stigmatization, with an OR of 4.75 (95% CI: 1.09–20.81, *P* = 0.039). Disclosure of HIV status significantly affects stigmatization, with disclosed individuals having an OR of 4.37 (95% CI: 1.41–13.57, *P* = 0.011). Additionally, individuals with an average monthly income of ₦50,000-₦100,000 are significantly less likely to experience stigmatization, with an OR of 0.03 (95% CI: 0.008–0.11, *P* < 0.001).

**Table 10:** Association of socio-demographic parameters of study subjects with discrimination (Logistic regression analysis) *n* = 400.

Variable	Discrimination	
	OR [95% CI]	*P* > z
**Age**		
> 20	Reference	
20–29	1.78(0.290562–10.88299)	0.533
30–39	0.70(0.178499–2.752831)	0.611
40–49	0.80(0.249451–2.565658)	0.707
> 50		
**Gender**		
Female	Reference	
Male	0.33(0.136237–0.789087)	0.013
**Marital status**		
single	Reference	
Married	1.57(0.417577–5.88339)	0.505
Divorced	5.34(0.944528–30.16217)	0.058
Widowed	0.76(0.084505–6.916386)	0.811
**Ethnicity**		
Yoruba	Reference	
Hausa	0.21(0.040554–1.105638)	0.066
Igbo	0.39(0.120352–1.278844)	0.121
Others	0.59(0.213736–1.615236)	0.303
**Religion**		
Christianity	Reference	
Islam	1.59(0.596543–4.232781)	0.354
**Level of Educational**		
None	Reference	
Primary		
Secondary	0.13(0.013472–1.167885)	0.068
Tertiary	0.12(0.012748–1.041251)	0.054
**Occupation**		
Civil servant	Reference	
Artificer	11.28(2.180583–58.30819)	0.004
Trader		
Student	0.50(0.201388–1.217385)	0.126
Others		
**Area of residence**		
Within Ikeja	Reference	
Outside Ikeja	0.35(0.132875–0.904878)	0.030
Outside Lagos	0.76(0.265375–2.194287)	0.616
**Status disclosure**		
Undisclosed	Reference	
Disclosed	1.81(0.750922–4.386543)	0.186
**Partner’s status**		
Positive	Reference	
Negative	0.11(0.029828–0.441713)	0.002
Unknown	0.72(0.203469–2.543071)	0.609
**Duration on ART**		
< 5 years	Reference	
>=5 Years	1.57(0.55778–4.428542)	0.392
**Average monthly Income(₦)**		
< 50,000	Reference	
50,000–100,000	1.28(0.516153–3.151279)	0.598
100,001–500,000		
> 500,000	0.13(0.032582–0.487093)	0.003

Table 10 highlights several statistically significant variables associated with discrimination. Males are less likely to experience discrimination with an odds ratio (OR) of 0.33 (95% CI: 0.14–0.79, *P* = 0.013). Occupation as an artificer significantly increases the likelihood of discrimination, with an OR of 11.28 (95% CI: 2.18–58.31, *P* = 0.004). Residing j Ikeja is associated with a decreased likelihood of discrimination, with an OR of 0.35 (95% CI: 0.13–0.90, *P* = 0.030). Having a partner with a negative HIV status significantly reduces the odds of discrimination, with an OR of 0.11 (95% CI: 0.03–0.44, *P* = 0.002). Finally, an average monthly income of over 500,000 also significantly lowers the likelihood of experiencing discrimination, with an OR of 0.13 (95% CI: 0.03–0.49, *P* = 0.003).

## Discussion

This study found that, while PLWHA in Ikeja are aware of HIV-related stigma, information is limited, and experiences with stigma and discrimination continue to limit health outcomes. The findings are consistent with previous Nigerian and worldwide research indicating that stigma in healthcare settings hinders care-seeking and undermines ART adherence^59,61,63,64^.

Healthcare provider attitude were a significant obstacle, with more than one-third of respondents reporting discriminatory treatment. This reflects a recurrent issue, as seen in research across Sub-Saharan Africa^65,66^, where negative provider attitudes worsen social exclusion and delay treatment^55,67–70^. Improving healthcare worker training is thus crucial for eliminating institutional stigma^71,72^ which echoes research demonstrating how HIV stigma infiltrates multiple areas of life, affecting the opportunities and well-being of PLWHAs^70^.

Socioeconomic status revealed as one of the most powerful indicators of stigma and discrimination^72–77^. Participants with lower incomes reported considerably increased probabilities of bad encounters, consistent with evidence suggesting that poverty increases sensitivity to social exclusion^75^. Education also performed a protective impact, highlighting the importance of focused awareness and empowerment programs^77^.

Additionally, the disclosure of HIV status constituted a contradiction^78^. While disclosure is necessary for receiving care and support, it also dramatically increased exposure to stigma in this study. This mirrors larger findings that PLWHA balance the benefits of openness with the danger of social rejection. It is critical to establish safe disclosure environments that are protected by law^79^.

Furthermore, gender disparities were more significant in discrimination than in stigma, with women being more likely to receive unjust treatment^80^. This is consistent with data that HIV-positive women frequently face a twofold burden of stigma related to both infection and gender roles^81^. Studies on self-stigma in HIV-positive communities in Abeokuta, Nigeria; Tamil Nadu, India, confirms the idea that women may internalize negative societal attitudes more thoroughly than men, as seen by the somewhat higher ratings among females^34,82,83^. Gender-sensitive techniques should be integrated into tailored interventions^84^.

Again, cultural and religious views influenced stigma experiences^85^, as some individuals reported being excluded from communal or religious activities in this study. Cultural norms that stigmatize drug use and premarital sexual activity, both of which are frequently (and often incorrectly) linked to HIV transmission, are the root cause of stigma especially among youth^86–88^ These cultural stigmas pose serious barriers to acceptance and support for PLWHA^89^.

Media representations were another source of bad perceptions. Media portrayals reinforce negative stereotypes and societal stigma^58,90^. These findings highlight the significance of community engagement and responsible media advocacy in addressing negative perceptions^91–95^.

Our results are consistent with Goffman’s theory that stigma is a process that discredits people and prevents them from fully engaging in society^57^. One example of what Goffman called “discredited identities” is the great frequency of performed stigma in families and healthcare facilities, when PLWHA are explicitly labeled and treated as inferior^57^. Similar to this, many respondents’ dread of disclosure demonstrates “felt” or expected stigma, which is in line with Goffman’s theory that conduct is influenced by the maintenance of a spoiled identity even when there isn’t overt discrimination^57^.

Furthermore, Goffman’s contention that stigma is socially constructed and context-dependent is supported by the socioeconomic and gender-related factors found in this study, which demonstrate how stigma is exacerbated by larger social injustices^96^. Crucially, using this theoretical perspective highlights the necessity of multi-level interventions that target institutional practices, society norms, and individual attitudes in order to eliminate the stigmatizing processes that limit the lives of PLWHA^57^.

Overall, stigma had a significant impact on mental health, social interactions, and ART adherence, demonstrating its function as a hidden driver of poor HIV outcomes^97^. A multidimensional response is required, including legislative changes, healthcare practitioner training, socioeconomic support, and ongoing public education^98^. By addressing both structural and social variables, Nigeria can minimize stigma and enhance health equity for PLWHA^99^.

Although stigma and discrimination against PLWHA have been reported in other Nigerian and African contexts^96,97,100–104^, this study makes a distinct contribution by providing one of the first facility-based assessments in Ikeja, Lagos State. Unlike national or rural-focused studies^85,87^, our findings capture the unique urban dynamics of Lagos, where population density, cultural diversity, and health system pressures create specific challenges for PLWHA. By examining a broad set of determinants, we identified income level, disclosure of HIV status, and occupation particularly among artisans as strong independent predictors of stigma and discrimination, findings not commonly emphasized in prior literature^89,95–97,100–104^. Importantly, this study quantified the adverse impacts of stigma on mental health and ART access, thereby strengthening evidence of its direct consequences for health outcomes^105^. These contributions provide contextual novelty and programmatic relevance, offering actionable insights for state-level policymakers, healthcare providers, and community stakeholders seeking to reduce stigma and improve HIV care in Lagos.

The study has several limitations. First, its cross-sectional design limits the ability to make causal conclusions between factors and stigma-related experiences. Second, self-reported data may be subject to recollection or social desirability bias, which could result in underreporting or overreporting of stigma events. Third, the study was conducted in chosen ART facilities in Ikeja, which may not completely depict PLWHA experiences in rural or community-based settings, limiting generalizability. Finally, relying solely on quantitative tools may not adequately investigate the nuanced, lived experiences of stigma that qualitative methods can capture.

Despite these limitations, the findings have significant policy and practice implications. The data show that socioeconomic position, disclosure, occupation, and gender have a considerable influence on stigma and discrimination, emphasizing the importance of targeted interventions^106^. Health policymakers in Lagos State should include stigma-reduction training in healthcare worker curricula, increase anti-discrimination enforcement in healthcare facilities, and expand psychosocial support services within ART programs. Community-level interventions, such as economic empowerment efforts for low-income PLWHA and gender-sensitive programming for women, are critical to reducing vulnerability^107^. Furthermore, building safe spaces for disclosure can help balance the benefits of openness with safeguards against undesirable outcomes^108^.

Building on these findings, future studies should use longitudinal designs to monitor stigma experiences over time and determine the causal relationships between disclosure, stigma, and health outcomes. Further research on the effects of culture and religion on stigma, especially in urban vs. rural contexts, requires qualitative studies. While comparative research among Nigerian states may offer a more comprehensive understanding of regional variations, intervention studies may assess the efficacy of certain stigma-reduction tactics, such as training healthcare professionals and socioeconomic empowerment initiatives.

## Conclusion

The study concludes that stigma and discrimination against PLWHAs are still pervasive in Ikeja and have an impact on their general well-being, social inclusion, and access to healthcare. The difficulties that PLWHAs confront are mostly caused by socioeconomic issues, stigmas associated with culture and religion, and unfavorable attitudes from healthcare professionals. Even though stigma-related problems are becoming more widely recognized, there is still a significant need for improved education and focused treatments to deal with these problems. The results are consistent with previous research, which emphasizes the necessity for a multifaceted strategy to counteract stigma and discrimination connected to HIV.

## Supplementary Information

Below is the link to the electronic supplementary material.

Supplementary Material 1

## Data Availability

The datasets used and/or analysed during the current study available from the corresponding author on reasonable request.

## Abbreviations

AIDS: Acquired immune deficiency syndrome
ART: Anti-Retroviral Therapy
ATC: Antiretroviral Therapy Centre
HAART: Highly Active Anti-Retroviral Therapy
HIV: Human immunodeficiency virus
NARHS: National AIDS and Reproductive Health Survey
PEPFAR: US President’s Emergency Plan for AIDS Relief
PLHIV: People Living with HIV
PLWHA: People Living With HIV/AIDS
PMTCT: Prevention of Mother to Child Transmission
