# Supplementary material for: Stigma, discrimination and associated determinants among people living with HIV/AIDS accessing Anti-Retroviral Therapy in Ikeja, Lagos state, Nigeria
Source: Sci Rep. 2026 Mar 14;16:13523. doi: 10.1038/s41598-026-37218-2 (PMC13121804; doi:10.1038/s41598-026-37218-2)
Supplement: Supplementary file 1 — Supplementary Material 1 [file 41598_2026_37218_MOESM1_ESM.pdf]

STIGMA, DISCRIMINATION AND ASSOCIATED DETERMINANTS AMONG PEOPLE LIVING WITH HIV/AIDS ACCESSING ANTI-RETROVIRAL THERAPY IN IKEJA, LAGOS STATE, NIGERIA

APPENDIX

APPENDIX I

Consent Form for Research

Title of Research Project: Stigma, Discrimination and Associated Determinants Among People Living With HIV/Aids Accessing Anti-Retroviral Therapy in Ikeja Lagos State Nigeria.

Principal Investigator: Gambo Sidi Ali

What you should know about this study
You are being asked to join a research study
This consent form explains the research study and your part in the study
Please ask questions at any time about anything you do not understand
Ask any member of the study team to explain any words or information in this informed consent that you do not understand

Purpose of Research Project:

This study aims to comprehensively explore the knowledge, prevalence, types and determinants of stigma and discrimination among people accessing Anti-retroviral Therapy (ART) in Ikeja, Lagos State, Nigeria.

Procedures:

You are required to answer some questions on the above-stated topic. This should take about 15-20 minutes of your time. Please try to be sincere brief and clear as possible in your contributions.

Risk/Discomfort:

You may feel uncomfortable with divulging personal information or expressing your feelings on the certain aspects of your life and health status. There will be no needle pricks or bloodletting for any tests to be done.

Anticipated Benefits:

Your participation in this study will significantly improve the knowledge base on the level of Stigma and discrimination experienced by HIV patients and its association with access to ART service.

This may serve as baseline reference for policy formulation, program planning, implementation, and evaluation towards improving anti-retroviral therapy among HIV patients in Lagos state and in Nigeria.

Voluntary Participation:

You are a volunteer. You have the right to change your mind, or decide not to participate at any point during the study. There are no penalty or loss of benefit if you decide to quit the study. You should ask the research assistant or principal investigator any question you may have about this research study. During the study, we will tell you if we learn any new information that might affect whether you wish to continue to be in the study.

Who do I call if I have questions or problems?

Call the Principal investigator (Mr. Gambo Sidi Ali), at 08052257611
What does your signature on this consent form mean?

Your signature on this form means:
You have been informed about this study's purpose, procedures, possible benefits, and risks
You have received a copy of this consent
You have been given the chance to ask question before you sign
You have been told that you can ask any question at any time
You have voluntarily agreed to be in this study
You are free to stop being in this study at any time
If you stop being in this study, you understand it will not in any way affect your treatment at the ART Clinic.
You have agreed to co-operate with Mr. Gambo Sidi Ali and the research staff and to tell them immediately if you experience any unexpected or unusual symptoms.
Please indicate your name (participant):
Signature or Mark of Participant:
Date:
Signature of Person obtaining consent: Date:
Witness to consent if participant is unable to read or write Date
Signed Copies of this consent form must be:
Retained on a file by the principal investigator
Given to the subject. This consent document is NOT valid without the Ethical Committee stamp of approval.

APPENDIX II

QUESTIONNAIRE

AHMADU BELLO UNIVERSITY, ZARIA, KADUNA STATE, NIGERIA.

DEPARTMENT OF PUBLIC HEALTH

QUESTIONNAIRE ON THE STIGMA DISCRIMINATION AND ASSOCIATED DETERMINANTS AMONG PEOPLE LIVING WITH HIV/AIDS ACCESSING ANTI-RETROVIRAL THERAPY IN IKEJA LOCAL GOVERNMENT AREA, LAGOS STATE NIGERIA

Dear respondent,

The attached interview guide is intended to elicit information on the above topic as a postgraduate student of the above-named Department and University. The study aims to identify determinants of stigma & discrimination among people living with HIV/AIDS (PLWHAs). Kindly note that your participation is voluntary and obtained information will not be used for reasons other than the purpose of this study.

We kindly request your cooperation and active participation to design training programs that will enhance knowledge and improve the quality of healthcare services.

Date: ……………………………….   Study ID number ………

Instruction: Kindly respond to the below questions as sincere as possible in the provided spaces by ticking (√).

Socio-demographic

Age as at last Birthday: ……………..yrs.

Gender: a) Male ( ) b) Female ( )

Marital Status: a) Single ( ) b) Married ( ) c) Divorced ( ) d) Separated ( )

Partner's HIV Status ( )a) Positive ( ) b) Negative ( ) c) Unknown ( ) d) Not Applicable ( )

Ethnicity: a) Yoruba ( ) b) Igbo ( ) c) Hausa ( ) c) others (specify)………………………..

Religion: a) Christianity ( ) b) Islam ( ) c) Traditional ( ) d) Others (specify)………………

Occupation: (a) unemployed ( ) (b) self-employed ( ) (c) Civil Servant ( ) (d) Artificer ( ) (e) Others (specify)…………

Socioeconomic status

Monthly Income: please specify…………..

Educational level: a) None ( ) b) Primary ( ) c) Secondary ( ) d) Tertiary ( )

Current Employment Status: a) Employed ( ) b) Unemployed ( )

Type of Employment: a) Government ( ) b) Private Self ( ) c) Private non-Self

Number of Dependents: Please Specify………..

Residence: a) Within the LGA ( ) b) Outside the LGA ( ) c) Outside the State

How long have you been on ART: Please Specify……….

Knowledge:

Are you aware of what stigma related to HIV/AIDS means?

a) Yes ( ) b) No ()

Have you received information regarding discrimination against PLWHAs in Ikeja?

a) Yes ( ) b) No ( )

Do you know about support services available to address stigma and discrimination faced by PLWHAs?

a) Yes ( ) b) No ( )

Have you participated in educational programs related to HIV/AIDS stigma and discrimination?

a) Yes ( ) b) No ( )

Are you aware that reducing stigma could improve healthcare access for PLWHAs?

a) Yes ( ) b) No ( )

Prevalence:

Have you ever been treated differently by healthcare providers due to your HIV status?

a) Yes ( ) b) No ( )

Have you encountered negative attitudes or behaviors from family or friends because of your HIV status?

a) Yes ( ) b) No ( )

Have you ever felt ashamed or judged because of your HIV status?

a) Yes ( ) b) No ( )

Do you believe there is widespread discrimination against PLWHAs in Ikeja?

a) Yes ( ) b) No ( )

Have you personally experienced verbal abuse or insults related to your HIV status?

a) Yes ( ) b) No ( )

Types and Sources of Stigma/Discrimination:

Have you experienced stigma while accessing healthcare services for HIV/AIDS?

a) Yes ( ) b) No ( )

Have you encountered discrimination in educational or workplace settings due to your HIV status?

a) Yes ( ) b) No ( )

Do you perceive media or societal attitudes contribute to stigma associated with HIV/AIDS?

a) Yes ( ) b) No ( )

How do religious or cultural beliefs influence the treatment of PLWHAs in your community?

a) Good ( ) b) Poor ( )

Have you witnessed instances where PLWHAs were excluded from community events due to stigma?

a) Yes ( ) b) No ( )

Determinants Assessment:

Do you believe poverty or socioeconomic status influences how PLWHAs are treated in Ikeja?

a) Yes ( ) b) No ( )

Have you experienced stigma or discrimination due to gender or sexual orientation along with your HIV status?

a) Yes ( ) b) No ( )

Do healthcare provider attitudes affect stigma faced by PLWHAs?

a) Yes ( ) b) No ( )

Do you think education level or awareness impacts how people treat PLWHAs in Ikeja?

a) Yes ( ) b) No ( )

Are political or governmental policies contributing to discrimination against PLWHAs?

a) Yes ( ) b) No ( )

Factors affecting Stigma and Discrimination

Do you perceive the community in Ikeja to hold negative attitudes towards PLWHAs?

a) Yes ( ) b) No ( )

Have you observed community-based programs aimed at reducing stigma against PLWHAs in Ikeja?

a) Yes ( ) b) No ( )

Do you believe media representations of HIV/AIDS contribute to negative perceptions and stigma?

a) Yes ( ) b) No ( )

Have you noticed media campaigns addressing HIV/AIDS stigma in Ikeja?

a) Yes ( ) b) No ( )

Have you personally encountered healthcare providers displaying stigma towards PLWHAs?

a) Yes ( ) b) No ( )

Do you think healthcare provider attitudes affect PLWHAs' willingness to seek healthcare services?

a) Yes ( ) b) No ( )

Do PLWHAs in Ikeja face challenges accessing ART and necessary healthcare services due to stigma?

a) Yes ( ) b) No ( )

Have you or others encountered barriers while accessing healthcare due to HIV/AIDS-related stigma?

a) Yes ( ) b) No ( )

Individual and Psychological Factors:

Disclosure of HIV Status:

Are PLWHAs in Ikeja comfortable disclosing their HIV status to others?

a) Yes ( ) b) No ( )

Does fear of stigma or discrimination influence the decision to disclose HIV status?

a) Yes ( ) b) No ( )

Mental Health Impact:

Have you observed changes in mental health or self-esteem among PLWHAs due to stigma?

a) Yes ( ) b) No ( )

Do you believe stigma significantly affects the mental well-being and social interactions of PLWHAs?

a) Yes ( ) b) No ( )

Socioeconomic Factors:

Poverty and Stigma:

Do you think poverty amplifies the stigma experienced by PLWHAs in Ikeja?

a) Yes ( ) b) No ( )

Have you noticed differences in the treatment of PLWHAs based on socioeconomic status?

a) Yes ( ) b) No ( )

Education and Awareness:

Can education and awareness programs substantially reduce stigma and discrimination related to HIV/AIDS?

a) Yes ( ) b) No ( )

Have educational campaigns in Ikeja shown any impact on reducing stigma against PLWHAs?

a) Yes ( ) b) No ( )

Have been gossiped about?

a) Yes b) No c) Don’t know

Verbally insulted/harassed or threatened

a) Yes b) No

Husband/spouse/other household member have been discriminated against?

a) Yes b) No c) Don’t know

Sexual rejection?

a) Yes b) No

Excluded from social gatherings?

a) Yes b) No

Discriminated against by other PLWHAs?

a) Yes b) No

Excluded from religious activities?

a) Yes b) No

THANKS FOR YOUR PARTICIPATION.
